# Women are Warmer but No Less Assertive than Men: Gender and Language on Facebook

**DOI:** 10.1371/journal.pone.0155885

**Published:** 2016-05-25

**Authors:** Gregory Park, David Bryce Yaden, H. Andrew Schwartz, Margaret L. Kern, Johannes C. Eichstaedt, Michael Kosinski, David Stillwell, Lyle H. Ungar, Martin E. P. Seligman

**Affiliations:** 1 Department of Psychology, University of Pennsylvania, Philadelphia, Pennsylvania, United States of America; 2 Computer Science Department, Stony Brook University, Stony Brook, New York, United States of America; 3 Graduate School of Education, University of Melbourne, Victoria, Australia; 4 Psychometrics Centre, University of Cambridge, Cambridge, United Kingdom; 5 Department of Computer and Information Science, University of Pennsylvania, Philadelphia, Pennsylvania, United States of America; University of Vermont, UNITED STATES

## Abstract

Using a large social media dataset and open-vocabulary methods from computational linguistics, we explored differences in language use across gender, affiliation, and assertiveness. In Study 1, we analyzed topics (groups of semantically similar words) across 10 million messages from over 52,000 Facebook users. Most language differed little across gender. However, topics most associated with self-identified female participants included friends, family, and social life, whereas topics most associated with self-identified male participants included swearing, anger, discussion of objects instead of people, and the use of argumentative language. In Study 2, we plotted male- and female-linked language topics along two interpersonal dimensions prevalent in gender research: affiliation and assertiveness. In a sample of over 15,000 Facebook users, we found substantial gender differences in the use of affiliative language and slight differences in assertive language. Language used more by self-identified females was interpersonally warmer, more compassionate, polite, and—contrary to previous findings—slightly more assertive in their language use, whereas language used more by self-identified males was colder, more hostile, and impersonal. Computational linguistic analysis combined with methods to automatically label topics offer means for testing psychological theories unobtrusively at large scale.

## Introduction

How do women and men use words differently? While language use typically differs minimally across self-reported gender, statistical models can accurately classify an author’s gender affiliation with accuracies exceeding 90% [1], suggesting that some differences do indeed exist. Black box statistical models, however, provide little insight into the psychological meaning of these gender differences. In this study, we combine techniques from computational linguistics with established psychological theory. Through an exploration of the language of over 68,000 participants, language analysis identified the linguistic features that most differentiate language used by either self-reported females or males.

### Gender-Linked Language

The study of gender differences in language has a long history that spans gender studies, psychology, linguistics, communication, and computational linguistics, among other fields. Investigating gender differences has been, at times, considered controversial [2, 3], although a consensus has emerged that gender remains an important variable worthy of scientific investigation (e.g., [4, 5, 6]. While language use varies only minimally across gender [7], algorithms capable of identifying female versus male authors with a high degree of accuracy (e.g., [8]) beg the question: what linguistic features account for these measurable gender differences?

Individual studies and meta-analytic reviews have found evidence for *gender-linked language features*, such as words, phrases, and sentence length, that are used consistently more by one gender than the other (*male-linked* if used more by men; *female-linked* if used more by women). In most studies, researchers have identified gender-linked features by comparing text samples from self-identified females and males, counting the frequencies of theoretically interesting features in each text (e.g., use of the first-person singular), comparing average frequencies across gender, and then interpreting results in terms of psychological theory [9, 10, 11].

For example, a meta-analysis conducted by Newman et al. [12] compared the language of men and women across 14,000 samples of text from a broad range of sources. Individuals’ writings were processed into word categories using the Linguistic Inquiry and Word Count tool (LIWC; [13]). The authors reported gender differences in 35 word categories, although most effect sizes were small by conventional standards (|*d*| ≤ .20; [14]). Men used more articles (e.g., “a”, “an”, “the”), quantifiers (e.g. “few” “many” “much”), and spatial words (e.g., “above”, “over”), were more likely to swear, and were more likely to discuss money- and occupational-related topics. Women used more personal pronouns, intensive adverbs (e.g., “really”, “very”, “so”), and emotion words, and were more likely to discuss family and social life. The differences were interpreted as reflecting a male tendency towards objects and impersonal topics and a female tendency towards psychological and social processes. Another line of research found similar gender-linked features [15, 16]. Across these empirical studies and literature reviews, male-linked features included directives (e.g., “do this.”), judgmental adjectives (e.g., “good”, “stupid”), and references to location and quantity, whereas female-linked features included hedging (“seems”, “maybe”, “kind of”), longer sentences, intensive adverbs (e.g., “so”, “really”), and references to emotions (e.g., “excited”, “happy”, “hurt”).

Mulac et al. [17] compared the magnitude of gender differences to that of two cultures speaking the same language, suggesting that these features reflect a male culture that is direct, succinct, status-oriented, and object-focused, and a female culture that is indirect, elaborate, and person-focused. These differences matter because they influence perceptions of an author’s interpersonal qualities. On the basis of language samples alone, judges blind to authors’ self-reported gender tended to rate females as nicer, more pleasant, and more intellectual, and rated males as stronger, louder, and more aggressive [18, 19].

Leaper and Ayres [20] summarized decades of research by organizing meta-analyses of gender-linked language around the interpersonal dimensions of affiliation and assertiveness. They defined assertive language as language used to influence, such as imperative statements, suggestions, criticisms, and disagreements. Affiliative language was defined as language affirming the speaker’s relationship with the listener, including statements of support, active understanding, agreement, and acknowledgment. The meta-analysis indicated that men used more assertive language and women used more affiliative language, but the sizes of these differences was moderated by methodological features of each study. For example, differences in assertiveness were most pronounced when participants were asked to discuss non-personal topics or to deliberate a specific issue.

**Fig 1:**
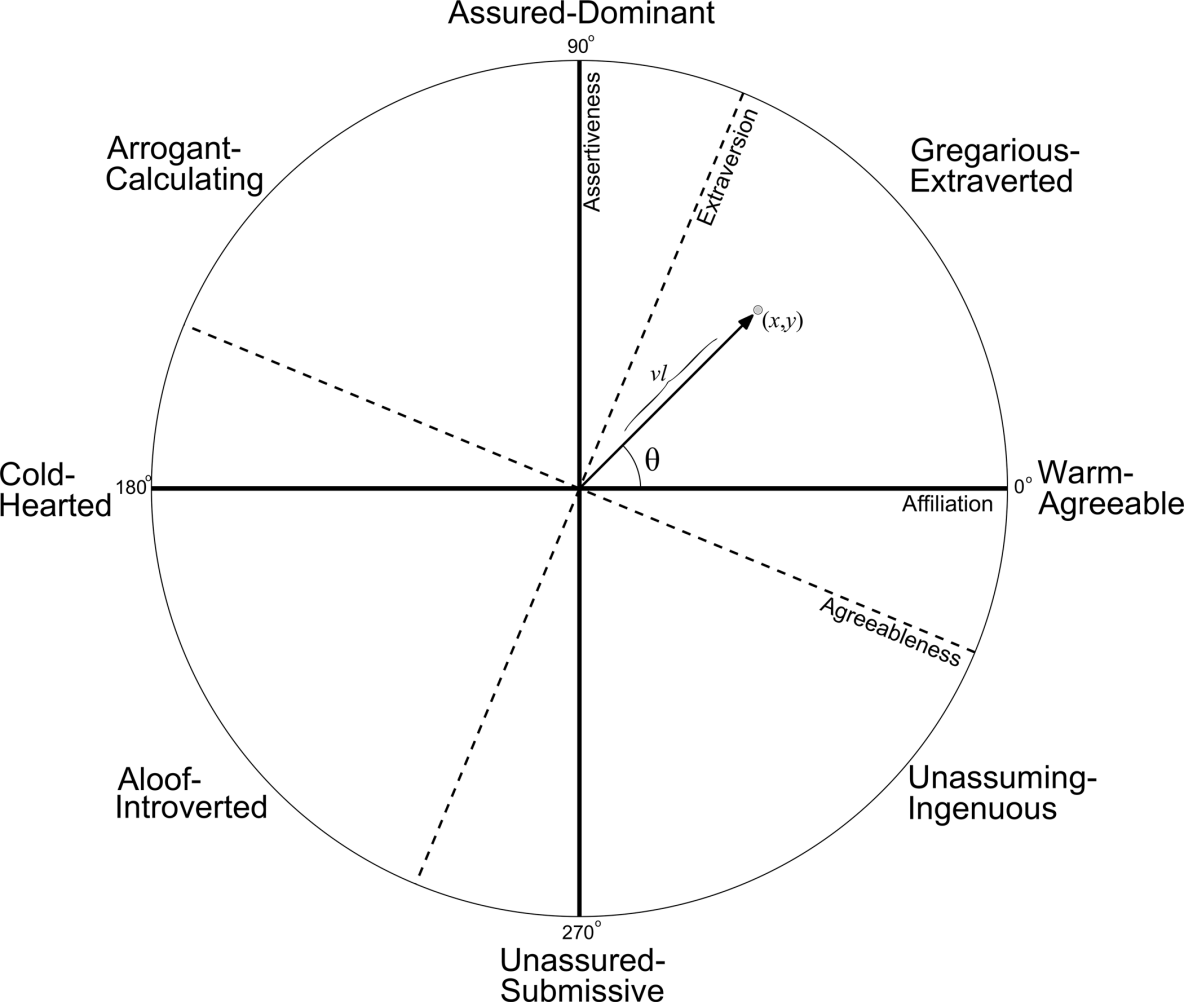
The Interpersonal Circumplex.

The prevalence of affiliation/assertiveness in gender research has motivated inquiry into how these dimensions relate to the Big Five personality framework. Assertiveness was found to correlate with extraversion, particularly the activity and excitement-seeking facets, whereas affiliation is captured by empathy-related aspects of agreeableness [21, 22]. Affiliation and assertiveness are the main axes of the interpersonal circumplex, a visual representation of behavioral tendencies (Fig 1) [23, 24]. The interpersonal circumplex is described in detail in Study 2, in which we demonstrate a method of automatically labeling topics as affiliative or assertive, based on personality scores of the people that use the topics most frequently.

### Closed vs. Open-Vocabulary Analysis

Most work on language differences by gender, including those above, have relied on *closed-vocabulary* analyses. These methods define categories of words *a priori*, based on common psychological or linguistic functions determined by researchers. The most popular implementation of closed-vocabulary analysis in psychology is LIWC, which automatically counts words belonging to over 60 predefined categories, such as *positive emotion* (e.g., “love”, “nice”, “sweet”), *achievement* (e.g., “earn”, “hero”, “win”), *articles* (e.g., “the”, “a”), and *tentative words* (e.g., “maybe”, “perhaps”, “guess”).

Closed-vocabulary methods depend on researchers at two levels: category definition and psychological labeling. Category definition refers to the creation of coherent groups of words, phrases, and other features (i.e., given a category, which words belong?). For example, word categories may be formed on the basis of a common syntactic function, such as first person singular words (e.g., “I”, “me”, “mine”) or prepositions (e.g., “in”, “on”, “with”), or by semantic content (e.g., positive emotion words such as “happy”, “joyful”, “excited”).

Psychological labeling refers to the process of inferring a category’s psychological meaning. Labeling is often done by the researcher or by trained raters and is often theory-driven. For example, Mulac [25] suggests that the frequency of using the first person singular is an index of a speaker’s emphasis on his/her own individuality. In the case of LIWC, the inferred psychological meaning of many word categories is implicit in their content (e.g., use of the *positive emotions* word category indicates a speaker’s experience of positive emotions) [26]. Such examples underscore the virtue of the theory-driven aspects of this approach. Other instances are less clear. For example, the language category *cognitive processes* is associated with having had a self-transcendent experience of unity, but the words most frequent within that category (“all”, “ever”, “every”) are likely references to a greater whole in this case, rather than indicators of a cognitive process [27]. Such discrepancies between category labels and the psychological meaning of the words that are most correlated with a given outcome introduce the potential for misleading interpretations of results.

Open-vocabulary methods of language analysis are newer within social science, but are common within computational linguistics and related disciplines [28]. These methods offer a data-driven alternative to the researcher-dependent category definition typically used in linguistic studies. Unlike closed-vocabulary methods, open-vocabulary methods use statistical and probabilistic techniques to identify relevant language patterns or topics. An example of an open-vocabulary method is topic modeling, which uses unsupervised clustering algorithms (i.e., latent Dirichlet allocation or LDA; [29]) to find potentially meaningful clusters of words in large samples of natural language (for an introduction to topic models, see [30]).

In a recent example, Schwartz et al. [31] applied LDA to a large collection of social media messages and identified 2,000 clusters of words, or *topics*. For example, one topic included the words “love”, “sister”, “friend”, “world”, “beautiful”, “precious”, and “sisters”, and a second topic included “government” “freedom”, “rights”, “country”, “thomas”, “political”, and “democracy”. These topics are generated in a data-driven, “bottom-up” way, as opposed to the theory-driven, “top-down” methods used in closed-vocabulary approaches.

Open-vocabulary methods may reveal new, unexpected patterns of gender similarities and differences. However, a challenge with language topics derived through open-vocabulary methods is how to infer their psychological meaning. Consider the two topics above: the first contains generally positive, relationship-related words, while the second appear to be words related to political discussions. The first topic has some salient social and emotional references, but the psychological meaning of the political topic is less clear. While we may have intuitions about the characteristics of the people who use each topic, the psychological meaning of a topic is not obvious. To this end, psychological theory can provide a framework for understanding and interpreting automatically derived topics.

In two studies, we examined gender language differences through an open-vocabulary analysis of language. In Study 1, we generated thousands of topics, and compared their relative use in a sample of over 52,000 female and male participants. This identified hundreds of male and female-linked topics. In Study 2, we labeled these gender-linked topics by degree of assertiveness and affiliation in a sample of over 15,000 people, and compared the pattern of gender-linked language along these two dimensions. Most studies require human raters to manually sort topics as either assertive or affiliative, whereas our method did so automatically. Further, while we and others have previously correlated topics with personality, here we use the correlations as labels. This labeling method places our open-vocabulary findings into a broader psychological context and allows comparisons with previous findings in the literature.

## Study 1: Identification of Gender-Linked Language

In Study 1, we used open-vocabulary methods to categorize a large set of language from social media into a smaller set of topics. By comparing the relative use of each topic across several thousand self-identified men and women, we identified *gender-linked topics*—topics used consistently more by one gender. One advantage of using social media as a language corpus is that it constitutes naturally occurring language among friends, family, and acquaintances. Only later—with users’ permission—was this language retrieved for research purposes. This allowed us to study language use in the relatively naturalistic setting of an online social networking site.

### Materials and Methods

Our language source was messages from Facebook, a popular social networking platform [32]. Participants were drawn from users of the MyPersonality application, a third-party Facebook application used by over four million people [33]. MyPersonality allowed users to complete several psychological measures, including many popular personality scales. All users provided written consent to the anonymous use of their responses for research purposes. In addition, a subset of these users allowed the application to access all of their past Facebook *status updates*. These participants also agreed to written informed consent within the MyPersonality application. An archival dataset of over 10 million users, collected between 2007 and 2012 is available for research use (the authors may be contacted at mypersonality.org) [34]. We use a subset of the available data here. As all language results are reported in aggregate, participants (including some minors) were exposed to minimal risk. The University of Pennsylvania’s Institutional Review Board approved all study procedures.

Status updates are a primary form of communication on the Facebook platform. These messages are typically visible to all first-degree friends in one’s social network. Status updates allow users to instantly broadcast information about themselves, such as current moods, activities, reactions, and relationships, to their social network. We created our analytic sample by selecting users who granted the MyPersonality application access to their status messages, wrote at least 1,000 words across their status messages, provided both their gender and age, and indicated that they were between 16 and 64 years of age, resulting in a final sample size of 68,228 participants for studies 1 and 2. Within this sample, 52,401 participants (64% female) were included in study 1, while the remaining 15,827 were set aside for study 2. Participants in this latter group were selected because they had completed a 100-item personality measure, while participants included in study 1 had not. The average user age was 26.2 years old (*Median* = 23, *SD* = 9.3, interquartile range = 20 to 29). Between January 2009 and November 2011, our final sample wrote approximately 15.4 million status messages. The average user (mean) wrote 4,077 words across all status messages (*Median* = 2,869; *SD =* 3,849.8).

Analyses comparing the 68,228 participants in studies 1 and 2 to the full sample of over 10 million participants revealed that study participants were significantly less extroverted (M = 3.41 vs. 3.56, d = -.19, 95% CI = -.21, -.18), and included more females than males (62.6% versus 50.9%). There were no significant differences in terms of age or the other personality characteristics.

#### Language analyses

As topic-based linguistic analyses of gender differences have rarely been done, we used this open-vocabulary approach to generate insights that complement and go beyond prior closed-vocabulary analyses. Prior to identifying topics, we first identified single words within the language sample. Words were defined by an emoticon-aware tokenizer [35], which identifies standard words, as well as language features more common in digital communication: emoticons (e.g., “:)”, “*^-^*”), non-standard punctuation (e.g., “!!!”), and unconventional spellings and acronyms (e.g., “feelin”, “lol”, “wtf”).

After extracting and tokenizing words and other language features, we used topics, derived via an unsupervised algorithm, latent Dirichlet allocation (LDA) [36], to define naturally-occurring groups of words. LDA uses Bayesian probabilistic modeling to identify clusters of words, or topics, that tend to co-occur within messages. LDA assumes that topics are mixtures of words and that documents (in this case, status updates) are mixtures of a fixed number of latent topics, which is specified by the analyst in advance. When applied to a set of messages, LDA identifies the words that define each topic along with their probability of occurring in the topic (i.e., a weight). Heavily weighted words are more prevalent within a given topic than less weighted words. We fit an LDA model using the Mallet package [37].

As the number of topics needs to be pre-specified, we set the number of topics to 2,000 to balance breadth and semantic coherence, and to be consistent with the precedent we set by using this number in our prior work [38]. The same word can belong to multiple LDA topics. This is a useful feature, as words have multiple parts-of-speech (e.g. “play the game” versus “went to the play”) and senses (e.g., crude oil versus crude person). However, this can result in cases in which two or more LDA topics overlap in their constituent words, creating semantically-similar topics with minimal differences. Automatically screening the topics, we found 719 redundant topics, also defined in this previous work, as those that shared more than 4 of their top 15 most heavily weighted words, resulting in a final set of 1,281 unique LDA topics.

A single topic consists of hundreds of words along with weights, but only a small handful of words have appreciable weights. We found that listing the most heavily weighted 5 to 10 words in order of decreasing weights is often sufficient to portray the semantic content captured by a given topic.

We then calculated the relative use of each topic for every user. Topic use for a given individual was defined as the probability of using a topic,

p(topic|user)=∑p(topic|word)×p(word|user)

where *p*(*word*|*user*) is the user’s normalized use of a word and *p*(*topic*|*word*) is the probability of the topic given that same word (which is part of the output of the fitted LDA model).

Lastly, we estimated the size of gender differences for all 1,281 topics using Cohen’s *d*, the standardized difference in group means, and 95% confidence intervals.

### Results

**Fig 2:**
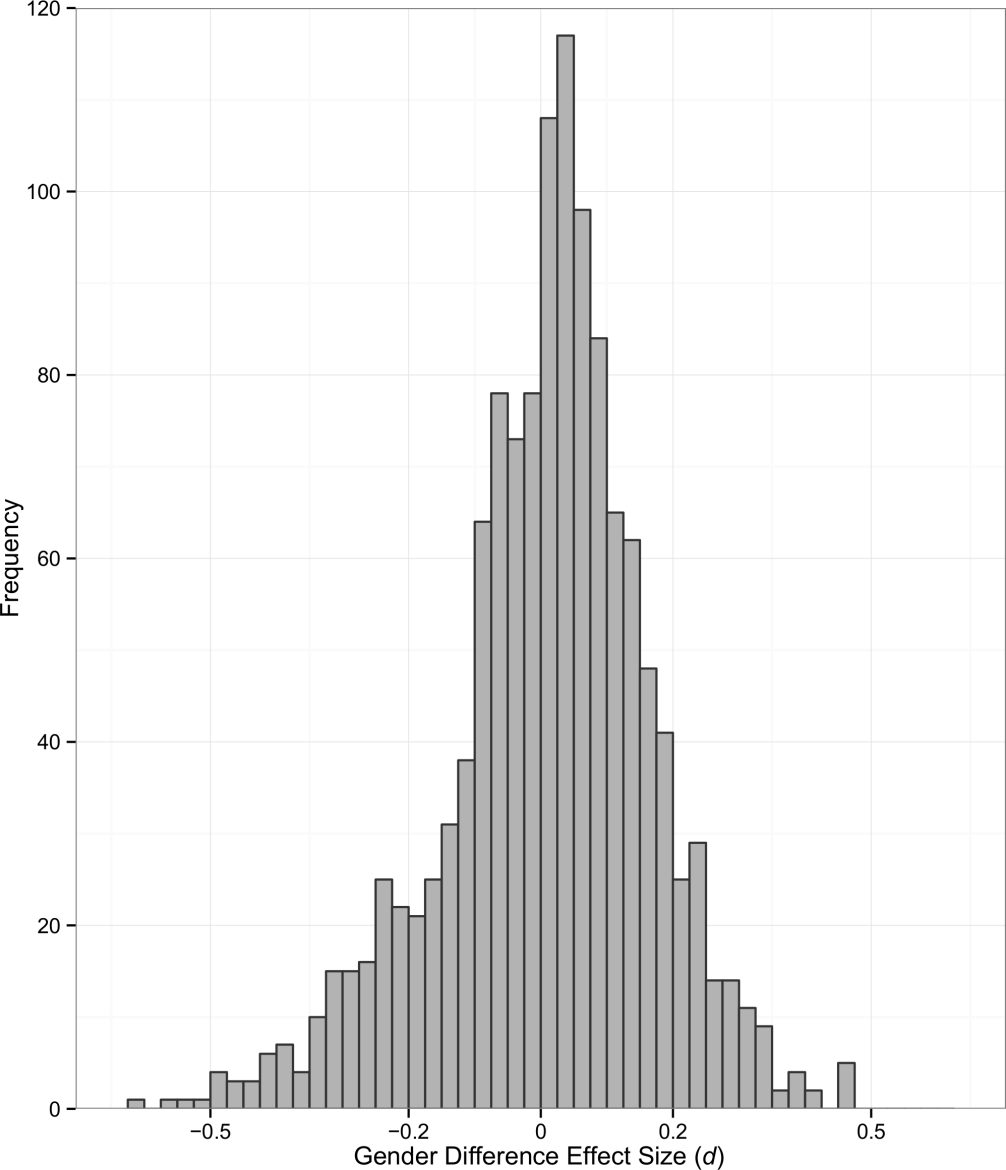
Gender Difference Effect Sizes. Effect sizes (Cohen’s *d*) of gender differences across 1,281 language topics.

We found hundreds of gender-linked language topics (i.e., topics that were used consistently more by one gender than the other). The average absolute effect size (|*d*|) across all 1,281 topics was 0.12. The full distribution of gender difference effect sizes is shown in Fig 2. Of 1,281 topics, 581 topics had absolute effect sizes (|*d*|) greater than 0.10; 250 had absolute effect sizes greater than 0.20. Only 5 topics reach the level of a “moderate” effect (|*d*| ≥ .5).

Tables 1 and 2 list the top 20 most female- and male-linked topics and their corresponding effect sizes (see Supplement 1 for full list of 1,281 topics and effect sizes). The most strongly female-linked topics included words describing positive emotions (e.g., “excited”, “happy”, “<3”, “love”,), social relationships (e.g., “friends”, “family”, “sister”), and intensive adverbs (e.g., “sooo”, “sooooo”, “ridiculously”). Strongly male-linked topics included words related to politics (e.g., “government”, “tax”, “political”), sports and competition (e.g., “football”, “season”, “win”, “battle”), and specific interests or activities, such as shooting guns, playing musical instruments, or playing video games. Note that topics are semantically-related clusters of words identified automatically by latent Dirichlet allocation. In Tables 1 and 2, Words are ranked in descending order of prevalence (weight) in each topic.

**Table 1:** Top 20 female-linked language topics and effect sizes.

Top female topics	*d*	95% CI
excited, tomorrow, tonight, soooo, sooo, super, yay, sooooo, uber, ridiculously	.63	(.61, .65)
happy, birthday, wishing, sister, years, wonderful, st, daughter, nephew, brother	.62	(.60, .64)
cute, baby, adorable, puppy, sooo, aww, soo, he's, soooo, awww	.55	(.53, .57)
<3,:), ily, babe, boyfriend, par, besties, bestie, =], xoxo	.53	(.51, .55)
family, friends, wonderful, blessed, amazing, thankful, loving, husband, grateful, lucky	.51	(.49, .53)
love, loved, truely, freely, shown, dearly	.50	(.48, .52)
:),: (, dayy, funn, soo, todayy, goood, alll, yayy	.49	(.47, .51)
shopping, christmas, grocery, clothes, xmas, online, shoppin, spree, lunch, mall	.48	(.46, .50)
love, sister, friend, world, beautiful, precious, sisters, thin, words, shared	.48	(.46, .49)
<3, brandon, zach, amazing, jr, robert, boyfriend, heather, katie, mummy	.47	(.45, .49)
love, yo, adore, xoxo, admire, extraordinary, absolutly, genuine, entitled, mentioned	.46	(.45, .48)
<3,: d,:), ;), amazing, xx,: p,: '), xxx, xxxx	.45	(.43, .47)
<3, love, ^_^, ^.^,; d, sweetheart, hun, inlove, mucho, girlies	.44	(.42, .46)
:), fun, haha, danielle, marie, hanging, sooo, soo, hahaha, hahah	.43	(.42, .45)
miss, dad, man, missing, girl, daddy, forever, boyfriend, show, husband	.43	(.41, .45)
sister, niece, nephew, loves, aunt, nieces, nephews, secrets, auntie, mother	.42	(.40, .44)
great, lunch, nice, dinner, family, enjoyed, church, wonderful, afternoon, sunday	.41	(.39, .43)
love, loving, jared, love's, haters, losers, enemies, secure, ing, supported	.41	(.39, .43)
day, wonderful, hope, great, blessed, beautiful, fantastic, filled, goodmorning, glorious	.41	(.39, .43)
house, family, mom, cousins, grandparents, dinner, cousin, grandma, parents, dad	.41	(.39, .43)

**Table 2:** Top 20 male-linked language topics and effect sizes.

Top male topics	*d*	95% CI
government, freedom, rights, country, thomas, political, democracy, liberty, america, power	-.47	(-.45, -.48)
win, lose, game, losing, winning, bet, loose, loses, streak, wi	-.47	(-.45, -.48)
battle, fight, victory, fighting, win, war, defeat, enemy, defeated, won	-.46	(-.45, -.48)
shit, holy, fuck, fucking, piece, bull, load, fuckin, ton, outta	-.45	(-.44, -.47)
football, team, season, game, play, players, league, sports, fantasy, player	-.45	(-.44, -.47)
metal, music, band, rock, bands, heavy, listening, singer, songs, listen	-.42	(-.40, -.43)
xbox, ps, play, playing, live, games, cod, online, wii, playin	-.41	(-.39, -.43)
opinion, opinions, logic, based, political, fact, moral, beliefs, philosophy, argument	-.40	(-.38, -.42)
world, cup, spain, win, england, fifa, germany, won, usa, final	-.40	(-.38, -.42)
government, economy, tax, budget, pay, taxes, country, income, benefits, obama	-.39	(-.37, -.41)
gun, guns, shot, shoot, shooting, range, barrel, loaded, shotgun, cracker	-.39	(-.37, -.41)
album, cd, listening, songs, song, release, listen, itunes, albums, released	-.37	(-.35, -.39)
music, sound, headphones, loud, bass, speakers, studio, hear, drum, volume	-.35	(-.33, -.37)
death, die, dies, died, painful, dying, loss, slow, cab, funeral	-.34	(-.33, -.36)
skills, management, business, learning, information, research,communication, engineering, development, technology	-.34	(-.32, -.36)
american, british, accent, history, asian, america, russia, country, western, african	-.34	(-.32, -.36)
computer, error, program, photoshop, server, properly, file, website, message, view	-.34	(-.32, -.36)
guitar, play, playing, hero, bass, drums, learn, learning, acoustic, electric	-.34	(-.32, -.35)
man, iron, chef, cave, flat, slave, tin, fist, properly, beg	-.34	(-.32, -.35)
kill, kills, killed, murder, killing, die, swear, dead, alive, boredom	-.34	(-.32, -.35)

### Discussion

Our open-vocabulary method revealed hundreds of gender-linked language topics. While most of the effect sizes were relatively small by conventional standards, each topic represents a dimension of the broader construct of language. Across hundreds of dimensions, these small differences can add up to create meaningful stylistic differences across gender.

We found several gender-linked topics that replicated earlier findings using closed-vocabulary methods or different language contexts. For example, the most female-linked topic included intensive adverbs (e.g, “soo”, “sooooo”, “ridiculously”), consistent with findings by Newman et al. [39] and Mulac [40]. Female-linked topics contained frequent references to social relationships, including types of relation (e.g., “sister”, “friend”, “boyfriend”) and associated emotions (e.g., “love”, “miss”, “thank you”). This is consistent also with Newman et al.’s finding that women were more likely to reference psychological and social processes.

In general, female-linked topics contained many more references to emotions than male-linked topics, replicating findings from earlier meta-analyses by Leaper and Ayres [41]. One advantage of the open-vocabulary method is the ability to capture these references even when they appear in unconventional or novel forms. For example, in addition to emotion words, several female-linked topics contained non-word emotional expressions, such as emoticon hearts (“<3”), smiles (e.g., “:)”, “^_^”), frowns (“:(“), and tears (“:’(“).

Our method also replicated several findings of male-linked language. For example, male-linked language included swearing and references to sports and occupations (e.g., “management”, “business”, “research” [42]). Notably, several of the male-linked topics were related to highly specific activities (e.g., video games, specific sports, listening to music) or groups of objects (e.g., computers, media devices), illustrating how our method captures a more granular level of detail than traditional approaches. Several topics included words related to potentially sensitive discussions: current events and politics (e.g., “government”, “obama”), death and violence (e.g., “killed”, “murder”, “death”), and general arguments (e.g., “opinion”, “logic”, “argument”). In contrast to female-linked language, the male-linked topics lacked reference to positive emotions or positive social relationships. Again, these findings converge with previous research, which found male-linked language to be impersonal and more object-focused [43, 44, 45].

The pattern of topics can also be viewed in light of people-focused versus object-focused language, as others have suggested. Meta-analyses have found that men had much stronger interests and preferences for working with things relative to people, whereas women showed the opposite pattern [46, 47]. Likewise, we found a strong tendency in men to talk about objects, whereas women talked more about people and social relationships. A similar objects-versus-people distinction emerged in Newman et al.’s [48] closed-vocabulary analysis of gender differences.

Although several of our open-vocabulary findings converged with previous work, our method also generated hundreds of gender-linked topics that did not fit neatly into earlier frameworks. For example, several of the politically-related male-linked topics (e.g., “government”, “rights”, “democracy”, and “taxes”, “obama”) are not easily categorized as objects- or people-oriented. In Study 2, we built on Study 1 by using a method to assign psychological labels to these topics and describe the pattern of gender differences along more psychologically meaningful dimensions relevant to the extant literature.

## Study 2: Interpersonal Patterns in Gender-Linked Language

Study 2 characterized gender-linked topics from Study 1 into meaningful psychological attributes. Our goal was to assess each topic according to dimensions that would be most relevant to past studies of gender language differences and also have broader psychological significance: affiliation and assertiveness.

### Affiliation, Assertiveness, and the Interpersonal Circumplex

Gender differences have often been characterized by at least one of two dimensions: (1) affiliation and interpersonal warmth versus impersonality and coldness, and (2) assertiveness and dominance versus indirectness and passivity. These two dimensions, which we call *affiliation* and *assertiveness*, are so common in language studies that Leaper and Ayres [49] organized their meta-analyses of gender language differences around these dimensions. Further, Newman et al.’s [50] summary of gender language differences as psychological and social processes versus object properties and impersonal topics aligns closely with the affiliation dimension. Assertiveness is also key dimension in the influential work of Lakoff [51]. Others have characterized men’s language as more assertive and direct and women’s as more polite and indirect [52].

The prominence of the dimensions of affiliation and assertiveness in language research follows a long history of describing interpersonal behavior and judgments along similar dichotomies: communion and agency [53, 54], love and dominance [55], nurturance and dominance [56], warmth and competence [57], valence and dominance [58], and compassion and assertiveness [59]. For simplicity, we refer to these dimensions as affiliation and assertiveness, but acknowledge that similar concepts have gone by many names.

Depue and Morrone-Strupinsky [60] described trait affiliation as a tendency towards “enjoying and valuing close interpersonal bonds and being warm and affectionate” (p. 314). In the Big Five framework, affiliation is captured by a blend of socially enthusiastic components of extraversion and the compassionate, empathetic components of agreeableness [61]. Following this, affiliative language should express empathy, warmth, and motivations to form or nurture interpersonal bonds.

Assertiveness reflects a tendency towards “dominance, ambition, mastery, and efficacy that is manifest in. . . interpersonal contexts” [62], p. 315). Items from trait scales of assertiveness include “I take charge” and “I see myself as a good leader” [63]. Within the Big Five framework, assertiveness closely relates to the facets of activity and excitement-seeking component of extraversion, and negatively correlates with the polite and modest components of agreeableness [64, 65]. Hence, assertive language should express motivation for social dominance, engagement, and activity, but not necessarily for the need to build or maintain interpersonal bonds.

Together, affiliation and assertiveness form the primary axes of the interpersonal circumplex (Fig 1), a rich system for describing interpersonal behaviors and measures [66]. A benefit of combining these into a two-dimensional system is the ease with which blends of the two dimensions can be expressed as locations in interpersonal space, either with traditional Cartesian coordinates (*x*, *y*) or polar coordinates (θ, vector length or *vl*). This space is often divided into distinct regions, each reflecting different interpersonal styles. The descriptive labels around the edge of the circumplex reflect the octants suggested by Wiggins [67]. For example, highly assertive and highly affiliative behaviors (or language) fall within the *gregarious-talkative* region, while highly assertive but highly unaffiliative behaviors fall within the *arrogant-calculating* region.

### Assigning Psychological Labels to Language

To determine the degree of affiliation and assertiveness of a given language feature, we considered the traits of the people who are most likely to use that language. That is, we reasoned that assertive language would be expressed disproportionately more often by people who scored high on measures of assertiveness. For example, if a language topic containing the words “family”, “friends”, “wonderful”, “blessed”, and “amazing” is used most frequently by people who are highly assertive and highly affiliative, then we label it as a highly assertive and highly affiliative language topic. Likewise, if the topic containing “computer”, “error”, “program”, “photoshop”, and “server” is used most by unassertive and unaffiliative people, then we label it as low on assertiveness and low on affiliation.

To derive these labels, we examined correlations between topic use and self-reported personality measures in a sample of over 15,000 Facebook users (separate from the sample used in Study 1). These users completed measures of extraversion and agreeableness–the two Big Five domains most relevant to the interpersonal circumplex [68, 69, 70]. Within the hierarchy of personality traits proposed by the Five Factor Model, affiliation is aligned with specific facets of agreeableness (altruism, trust, and tender-mindedness), and assertiveness is aligned with specific facets of extraversion (assertiveness and excitement-seeking). DeYoung et al. [71] explicitly tested this model of affiliation and assertiveness across three samples and identified a good fit with extraversion and agreeableness (at approximately 67.5° and 337.5°, respectively). We follow these calculated angles and approach in our analyses.

We built on these findings by first calculating the correlations between each topic and facets of extraversion and agreeableness, and then rotating these (see “Affiliation, Assertiveness, and the Interpersonal Circumplex” above) to determine topic correlations with affiliation and assertiveness. This allowed us to plot each topic in the circumplex, examine topics along each dimension, and compare the broader pattern of gender-linked topics within interpersonal space.

### Materials and Methods

#### Participants

Participants were users of MyPersonality who granted the application access to their status messages, wrote at least 1,000 words across their status messages, provided their gender and age, indicated that they were between 16 and 64 years of age, completed a 100-item personality measure, and were not a part of the Study 1 sample. Our resulting sample size was 15,827 individuals (57% female). The average participant’s age was 24.9 (*Median* = 22, *SD* = 8.2, interquartile range = 20 to 27).

#### Language data

Similar to Study 1, all language data was drawn from Facebook status messages. We applied the same fitted topics from Study 1, totaling 1,281 topics, to this second set of language data.

#### Measures

Participants completed a 100-item Big Five measure, which consisted of items from the International Personality Item Pool (IPIP) [72, 73]. This measure is similar to the 100-item NEO-PI-R [74] and contains 20-item subscales assessing each Big Five domain. We used the participants’ scores on the 20-item Extraversion and Agreeableness scales as measures of these respective traits.

#### Affiliative and assertive topic labeling

To determine the topic’s degree of affiliation and assertiveness, we first estimated each topic’s correlations with extraversion and agreeableness, controlling for age and gender. Controlling for age and gender ensured that our resulting labels did not merely reflect gender differences in underlying personality trait distributions. Because extraversion and agreeableness were correlated in our sample (*r* = .24), we controlled for each trait when calculating correlations for every topic. We standardized topic use, extraversion, and agreeableness scores across users. Then, we regressed topic use on extraversion, agreeableness, gender, and age. The resulting regression coefficient for extraversion is equivalent to a Pearson correlation between the topic and extraversion, controlled for agreeableness, gender, and age, and the resulting regression coefficient for agreeableness is equivalent to a Pearson correlation between the topic and agreeableness, controlled for extraversion, gender, and age

Each topic’s correlations with extraversion and agreeableness were then used to create affiliation and assertiveness scores, which also determine its position in the interpersonal circumplex in Cartesian (*x*, *y*) coordinates, a process used in other studies utilizing circumplex models [75]. Within the classic interpersonal circumplex model, affiliation is located at 0° and assertiveness is located at 90°. Following precedent [76], we assumed that our measures of extraversion and agreeableness were located at 67.5° and 337.5°, respectively. By using topic correlations with agreeableness and extraversion as loadings on each respective dimension, we calculated a topic’s corresponding loading on affiliation and assertiveness using

$${affiliation}_{topic}=x_{topic}=\cos(67.5)\times r_{ext}+\cos(337.5)\times r_{agr}$$

$${assertiveness}_{topic}=y_{topic}=\sin\left(67.5\right)\times r_{ext}+\sin\left(337.5\right)\times r_{agr}$$

where (*x*_*topic*_, *y*_*topic*_) are a topic’s loadings on affiliation and assertiveness, respectively, and *r*_ext_ and *r*_agr_ are a topic’s correlations with extraversion and agreeableness, respectively. Thus, *affiliation_topic* and *assertiveness_topic* contain affiliation and assertiveness effect sizes for the given topic, which can be plotted within a two-dimensional plane where affiliation is the x-axis and assertiveness is the y-axis.

### Topic Analysis

#### Affiliation, assertiveness, and gender difference effect sizes

After labeling topics by affiliation and assertiveness, we analyzed the pattern of gender differences across each dimension. We first created scatterplots to compare the gender difference effect (*d*) of topics to their respective level of affiliation and assertiveness, and we calculated the Pearson correlation between *d*s (i.e., the extent to which the topic was used by females) and each dimension. We also examined the language content of topics near the tails of each dimension to assess whether our automatic labels identified reasonably assertive and affiliative language, or their opposites (described above) deferential and cold-hearted, respectively.

#### Gender-linked topics in the interpersonal circumplex

To focus specifically on patterns of gender-linked topics identified in Study 1, we limited our analysis to topics that had non-trivial gender differences, which we defined as those with |*d*| ≥ .05. Alternatively, we could have used the gender difference effect sizes (*d*s) across topics as estimated in the sample of 15,827 participants who also completed the 100-item personality questionnaire. We calculated these *d*s, too, and across both samples the *d*s were correlated at *r* = .98. We opted to use the *d*s from the Study 1’s sample of 52,401 participants due to the much larger sample size, but the pattern of results would not have meaningfully changed had we used gender difference *d*s from Study 2. Of the 1,281 topics, 905 met this criterion. We then used affiliation and assertiveness labels to place each gender-linked topic into the interpersonal circumplex, and explored the spatial distribution of these topics in two complementary ways. First, we visualized the pattern of differences by plotting topics in the circumplex and shading the corresponding points according to the size and direction of the gender difference. This visualization offers an overview of the differences across hundreds of topics.

Second, we compared the distributions of male- and female-linked topics within interpersonally distinct areas of the circumplex octants. Octants are formed around the primary and secondary axes, and we use the divisions and labels suggested by Wiggins [77]. Within each octant, we counted the total number of gender-linked topics, the proportion of those topics that were female-linked, and determined mean and median *d* of all gender-linked topics.

#### Group summaries

Finally, we summarized the central tendency and variability of each group of topics (comparing male-linked and female-linked *topics*, not male and female participants). We visualized these summaries using iconic representations, which illustrate group differences within a circumplex space. To produce iconic representations, we calculated the mean angle or *circular mean* (θ_*M*_) within each group of topics. A group’s circular mean describes its “predominate theme” or “center of gravity,” p. 417 [78]. To calculate each group’s circular mean, we first calculated the angular position (θ_*topic*_) of every topic as

θtopic=tan−1(ytopicxtopic)

Here, θ_*topic*_ describes the interpersonal style or flavor of an individual topic (e.g., 0° = warm, agreeable, compassionate; 135° = cold, arrogant, calculating). We next used each θ_*topic*_ to locate each topic along the circumference of the unit circle, or (*x’*, *y’*), as

$${x\prime }_{topic}=\cos\left(\theta_{topic}\right),$$

$${y\prime }_{topic}=\sin\left(\theta_{topic}\right)$$

We calculated each group’s mean *x* and *y*, or *x*_*M*_ and *y*_*M*_ by averaging across the individual *x’*_*topic*_ and *y’*_*topic*_, respectively. Each group’s circular mean, θ_*M*_, is the angle corresponding to their respective *x*_*M*_ and *y*_*M*_, or

$$\theta_{M}=\tan^{-1}\left(y_{M}/x_{M}\right).$$

To summarize the variability of each group of topics, we calculated the circular variance within each group as

$$var(\theta )=1-\frac{\sum \cos\left(\theta_{M}-\theta_{topic}\right)}{n},$$

where *n* is the number of topics in each group. We then converted this to degrees and plotted ±1 unit of variance around each group mean. Finally, we estimated 95% confidence intervals around each group’s θ_*M*_ using the approximation suggested by Gurtman & Pincus (2003),

$$95\%\;CI=\theta_{M}\pm 1.96\times \frac{\cos^{-1}\left(1-var(\theta )\right)}{\sqrt{n}}.$$

The resulting iconic representations display the circular mean (as arrows), corresponding confidence intervals around the mean (as dark shading), and ±1 unit of variance around each mean (as light shading).

### Results

Our method automatically labeled topics by assertiveness and affiliation, and patterns of male- and female-linked language reflected contrasting interpersonal styles. Highly affiliative language was used much more by female participants. Gender differences in the use of assertive language were less clear, though women used slightly more assertive language.

#### Affiliation and assertiveness topics across gender

The most affiliative topics (Table 3) were centered on positive social relationships, positive emotions, and positive evaluations; the least affiliative topics contained swear words, negative evaluations of others, and argumentative language. The most assertive topics (Table 4) contained language related to intense social engagement (e.g., “party”, “dance”, “rave”, “club”), excitement seeking, and engaging one’s network (e.g., “wanna”, “holla”, “lets”, and a topic of first names); the least assertive topics contained references to working with computers, book reading, uncertainty (e.g., “suppose”, “strange”, “sort”, “unpredictable”), and waiting (e.g., “time”, “waiting”, “long”).

**Table 3:** Affiliation Topics. Top 10 topics with highest and lowest affiliation scores.

Topics	affiliation
family, friends, wonderful, blessed, amazing, thankful, loving, husband, grateful, lucky	.16
day, wonderful, hope, great, blessed, beautiful, fantastic, filled, goodmorning, glorious	.14
day, great, today, hope, remember, begins, quickly, catch, reach, calling	.13
friends, family, fb, dear, hang, closest, close, misses, hanging, catching	.11
great, night, time, fantastic, catching, evening, lastnight, workout,: o, topped	.11
great, lunch, nice, dinner, family, enjoyed, church, wonderful, afternoon, sunday	.11
great, job, guys, amazing, crew, awesome, show, cast, proud, congrats	.10
beautiful, love, girl, wonderful, loving, daughter, girlfriend, woman, sweet, perfect	.10
today, made, awesome, rice, making, treats, equals, cakes,	.10
weekend, awesome, amazing, great, retreat, couples, cheers, buds, flippin,	.10
death, die, dies, died, painful, dying, loss, slow, cab, funeral	-.07
knife, cut, sharp, daily, stab, regular, basis, spoon, sword, blade	-.07
god, damn, swear, fucking, dammit, damnit, puff, powder, damned, dam	-.08
screw, screwed, damn, pissed, messed, totally, sucks, freaking, >:, crap	-.08
hate, haters, hating, jealous, hater, bitches, mad, hated, hatin, jealousy	-.08
idiot, loser, biggest, complete, total, losers, thinks, idiots, jerk, winner	-.08
opinion, opinions, logic, based, political, fact, moral, beliefs, philosophy, argument	-.08
kill, kills, killed, murder, killing, die, swear, dead, alive, boredom	-.10
people, rude, selfish, stupid, ignorant, assholes, arrogant, they're, pathetic, racist	-.11
shit, holy, fuck, fucking, piece, bull, load, fuckin, ton, outta	-.11

**Table 4:** Assertiveness Topics. Top 10 topics with highest and lowest assertiveness scores.

Topic	assertiveness
tonight, party, gonna, partying, rockin, town, poppin, club, messy, homies	.13
party, tonight, dance, lets, halloween, rave, tonite, slumber, bday, wasted	.13
love, yo, adore, xoxo, admire, extraordinary, absolutly, genuine, entitled, mentioned	.11
dj, party, free, club, drinks, pm, drink, lounge, ladies, bar	.11
ya, wanna, holla, gotta, yo, boi, hit, lemme, hey, ill	.11
girls, guys, guy, girl, boys, jerks, flirt, girlfriends, golden, boyfriends	.11
sarah, marie, emily, nicole, elizabeth, katie, taylor, lauren, johnson, ashley	.10
lil, lol, sis, big, cousin, bro, cuz, wit, sister, luv	.10
baby, girl, boy, beautiful, cousin, she's, congrats, sister, proud, meet	.10
im, goin, tired, rite, pissed, feelin, idk, gettin, confused, wont	.10
it's, time, late, turn, begin, knowing, apologize, limited, o'clock, earth	-.09
it's, time, long, i've, waiting, unpredictable, stays, reach,	-.09
internet, connection, cable, wireless, net, computer, slow, laptop, access, connect	-.10
isn't, it's, doesn't, anymore, natural, supposed, working, aren't, ironic, helping	-.10
today, didn't, earlier, yesterday, couldn't, ended, planned, expected, usual, started	-.11
computer, windows, laptop, pc, drive, gb, mac, files, installed, virus	-.11
anime, manga, bleach, episode, series, japanese, episodes, chapter, characters, ep	-.11
it's, thing, sort, isn't, odd, suppose, strange, thinks, apparently, unusual	-.11
books, read, book, reading, library, sees, bible, open, carry, comic	-.11
computer, error, program, photoshop, server, properly, file, website, message, view	-.11

**Fig 3:**
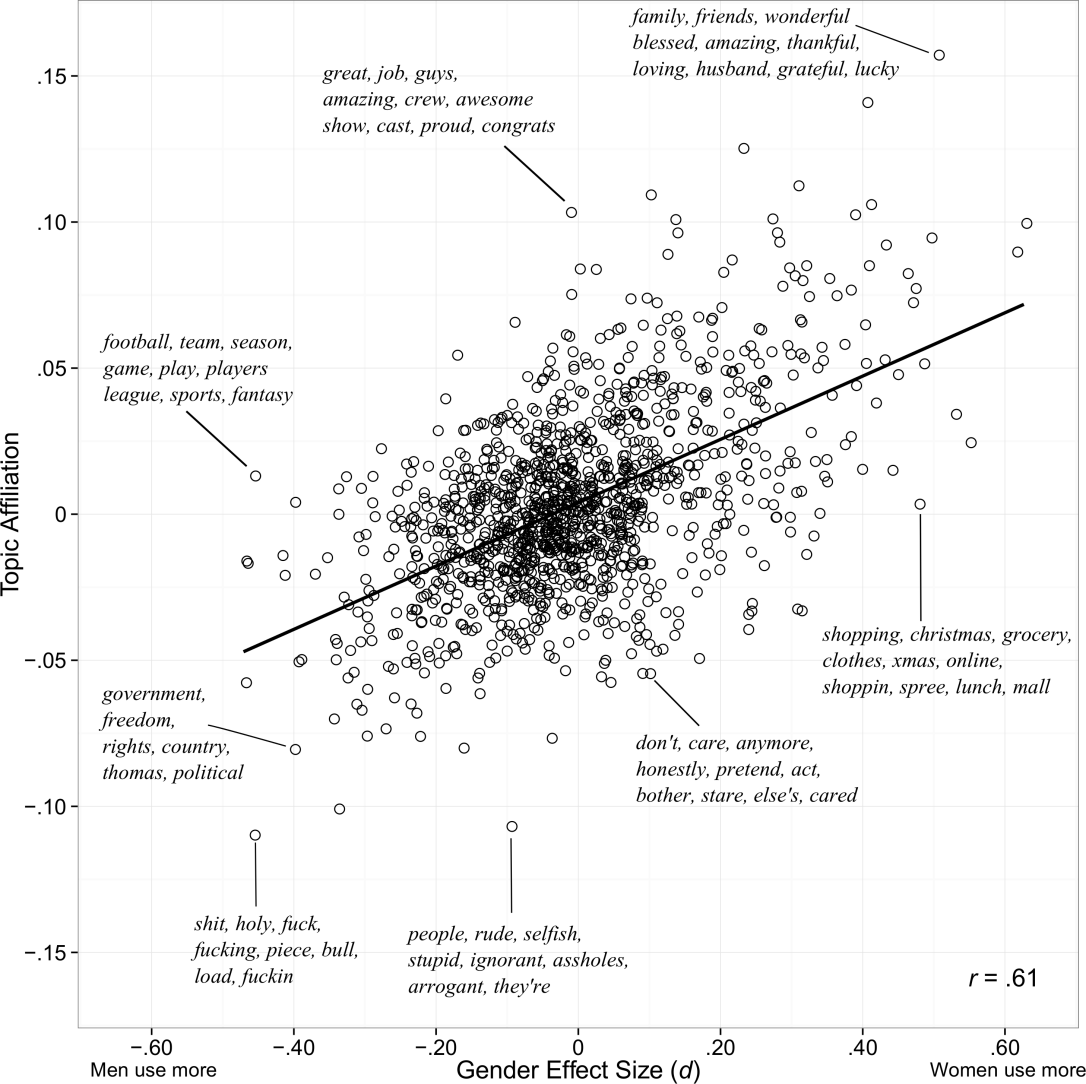
Affiliative Topics and Gender Effect Size. Language topics plotted against level of affiliation and gender difference effect size (Cohen’s *d*). For select topics, the most heavily weighted words are displayed. The black line is the best fitting line.

Topic affiliation score (i.e. *affiliation*_*topic*_) was positively correlated (*r* = .61, *p <* .001) to topic gender score (i.e. Cohen’s *d* between the topic and gender). Fig 3 plots individual topics by affiliation and gender difference effect size, and the words of several topics are listed to illustrate how content shifts across the range of both variables. Topics describing gender-typical activities (such as sports for men and shopping for women) had large gender effect sizes but virtually no loading on affiliation. Other topics had relatively high and low loadings of affiliation but no gender difference. For example, a topic including the words “great”, “job”, “guys”, and “amazing” was highly affiliative but was used equally by men and women.

**Fig 4:**
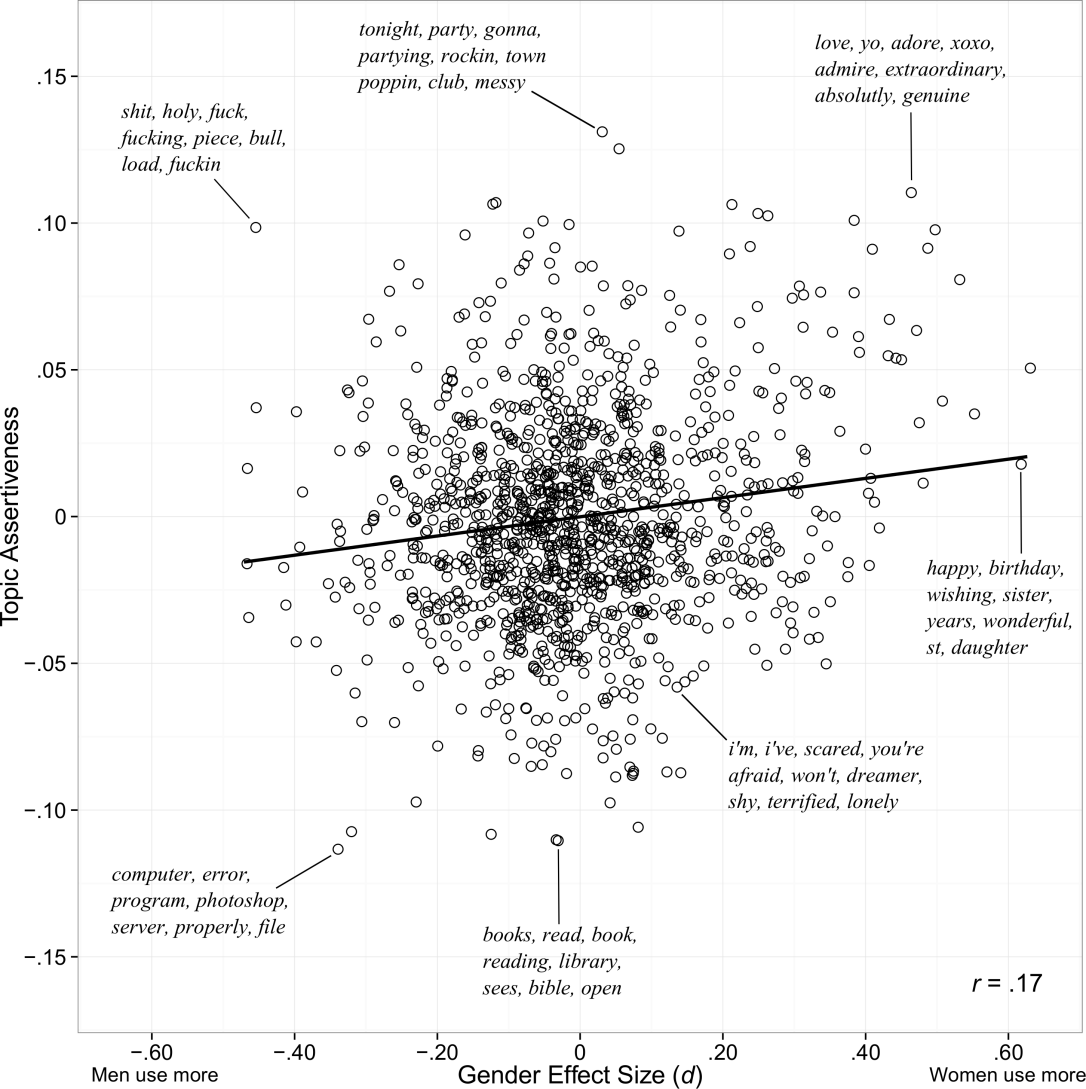
Assertiveness Topics and Gender Effect Size. Language topics plotted against level of assertiveness and gender difference effect size (Cohen’s *d*). For select topics, the most heavily weighted words are displayed. The black line is the best fitting line.

Topic assertiveness score (i.e. *assertiveness*_*topic*_) was positively correlated with topic gender score (*r* = .17, *p* < .001), but examination of the scatterplot in Fig 4 suggests that this correlation is driven largely by a small number of strongly female-linked and highly assertive topics; these topics contain words expressing positive emotion (e.g., “love”, “amazing”, “wonderful”). While all strongly female-linked topics had positive loadings on assertiveness, strongly male-linked topics were spread evenly across the assertive dimension. Male-linked topics high on assertiveness included swearing and critical language; male-linked topics low on assertiveness described objects and impersonal topics.

#### Gender-linked topics in the interpersonal circumplex

**Fig 5:**
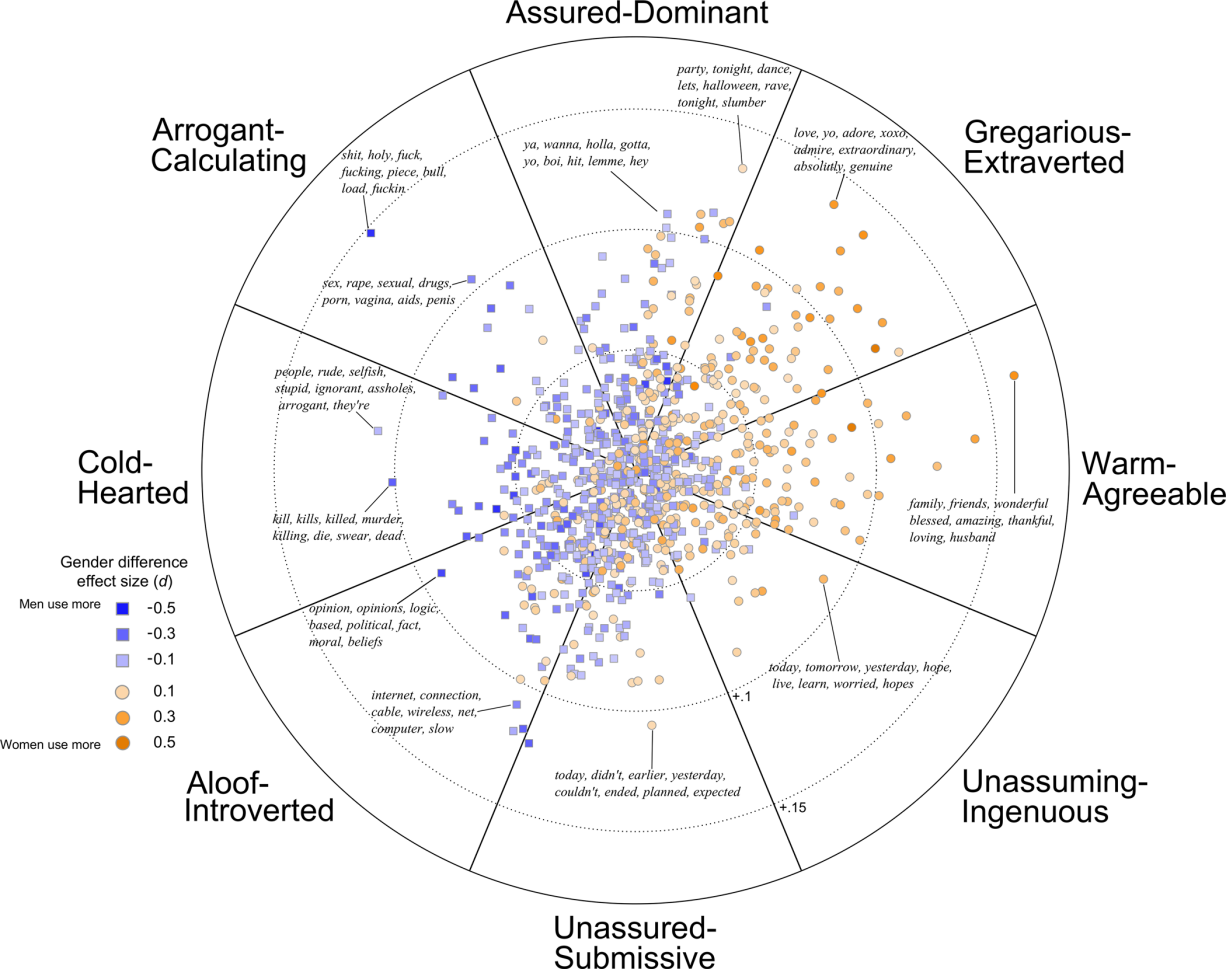
Gender-linked language topics in the interpersonal circumplex. Points are topics (semantically-related clusters of words.) Squares are topics used more by males; circles are topics used more by women. The color saturation of each point indicates the size of the gender difference effect size (Cohen’s *d*), with darker colors indicating stronger effects. The position of each topic is determined by its correlation with extraversion and agreeableness.

Fig 5 visualizes hundreds of topics and their corresponding gender differences effect sizes, highlighting words within select topics around the circumplex. Comparisons between topics’ words and their location in the circumplex suggest that this method accurately matches topics to their blend of assertiveness and affiliation. For example, many topics in the gregarious-extraverted octant (blending high assertiveness and high affiliation) contain enthusiastic expressions of positive emotion, often related to social relationships. In contrast, topics in the aloof-extraverted octant (blending low assertiveness and low affiliation) contain words referencing objects (e.g., computers and related technical words) and less social activities (e.g., film- and music-related terms).

The distinct pattern of female- and male-linked topics within the circumplex illustrates contrasting interpersonal styles. Overall, female-linked topics were more affiliative, but differences in assertiveness were more complex. While female-linked topics dominated the more affiliative half of the circumplex, they were also concentrated in the more assertive quartile (the warm-agreeable and gregarious-extraverted octants). Male-linked topics were largely in the less affiliative, colder half, but also spread more evenly in terms of assertiveness. Male-linked topics were both the most assertive (a swear word topic) and least assertive (topics with computer-related words).

Octant-level analyses of gender-linked topics and effect sizes were consistent with a pattern of greater affiliation across female-linked topics but greater variation in assertiveness across male-linked topics. Table 5 lists example topics from each octant (selected by having the longest vector length, or distance from the origin), the number of gender-linked topics within each octant, and the corresponding proportion that were female-linked. For example, of all the gender-linked topics within the gregarious-extraverted octant, 73% were female-linked; within the cold-hearted octant, only 11% were female-linked. Throughout the circumplex, more affiliative octants had greater proportions of female-linked topics. However, both the most assertive (assured-dominant) and least assertive (unassured-submissive) octants had relatively more male-linked topics. A similar pattern emerged from octant-level summaries of effect sizes. In more affiliative octants, mean and median *d*s favored women; in the most and least assertive octants, mean and median *d*s favored men.

**Table 5:** Gender similarities and differences within regions of the interpersonal circumplex.

Octants	Example topic (gender effect size, *d*)	Count of gender-linked topics	Proportion of female-linked topics in octant	mean *d*	median *d*
Warm- Agreeable	family, friends, wonderful, blessed, amazing, thankful, loving, husband, grateful, lucky (.51)	130	68%	.10	.12
Gregarious- Extraverted	love, yo, adore, xoxo, admire, extraordinary, absolutly, genuine, entitled, mentioned (.46)	124	73%	.12	.11
Assured- Dominant	party, tonight, dance, lets, halloween, rave, tonite, slumber, bday, wasted (.05)	128	38%	-.04	-.08
Arrogant- Calculating	shit, holy, fuck, fucking, piece, bull, load, fuckin, ton, outta (-.45)	63	11%	-.12	-.12
Cold-Hearted	people, rude, selfish, stupid, ignorant, assholes, arrogant, they're, pathetic, racist (-.09)	82	17%	-.12	-.14
Aloof- Introverted	anime, manga, bleach, episode, series, japanese, episodes, chapter, characters, ep (-.12)	162	23%	-.10	-.12
Unassured- Submissive	computer, error, program, photoshop, server, properly, file, website, message, view (-.34)	128	38%	-.03	-.07
Unassuming- Ingenuous	today, tomorrow, yesterday, hope, live, learn, worried, hopes, promised, goodbye (.29)	88	66%	.06	.08

**Fig 6:**
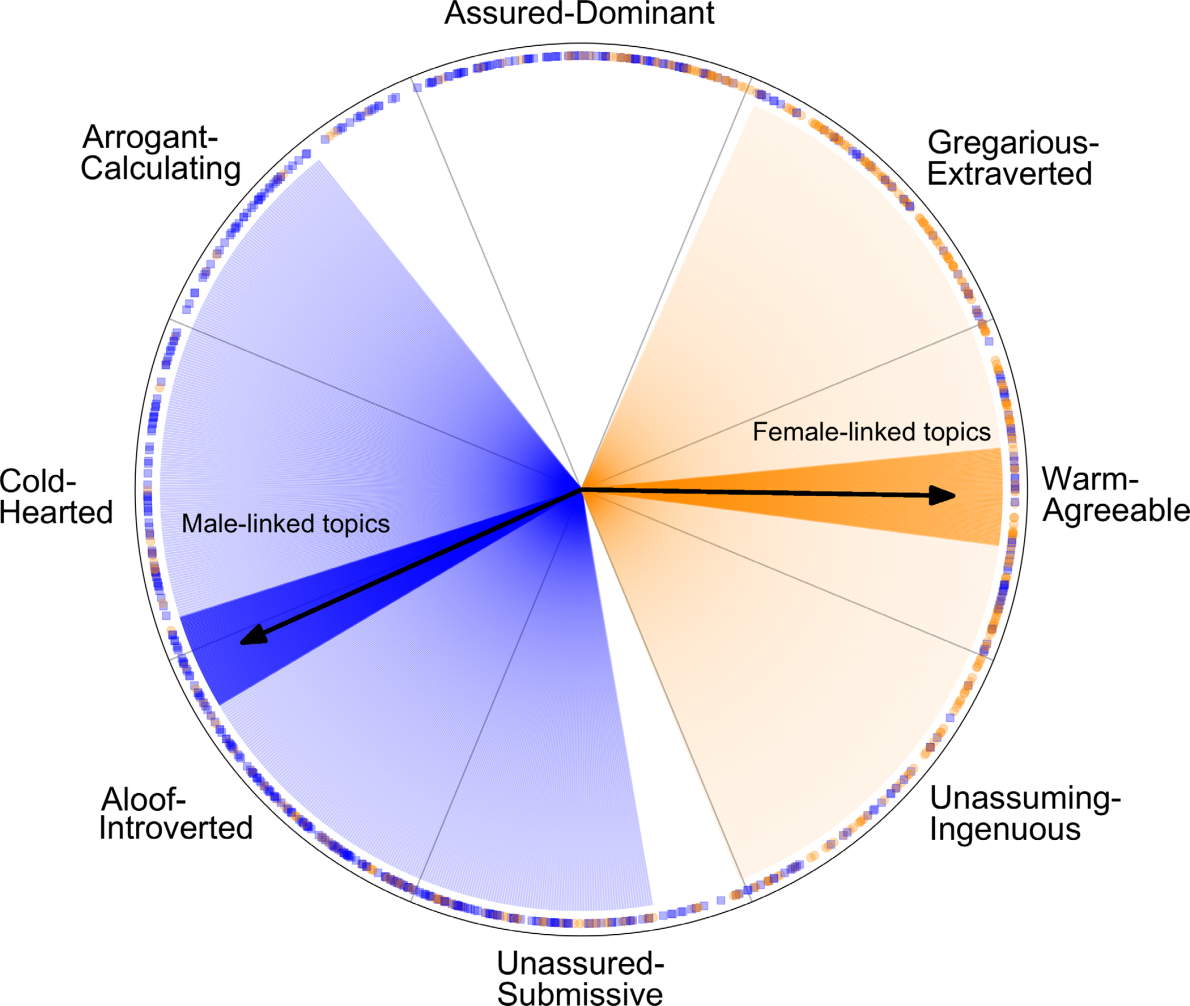
Iconic representation of gender-linked language topics. Arrows indicate the mean circular angle of male- and female-linked topics. Dark shaded bands around mean indicate 95% confidence intervals. Light shaded areas are defined by the mean angle +/- one standard deviation. Around the border of the circumplex, individual topics are plotted by angle. Male-linked topics are blue squares; female-linked topics are orange circles.

The mean angles of gender-linked topics, displayed as arrows in Fig 6, indicate that male- and female-linked topics, on average, reflected opposing interpersonal styles. The circular mean of male-linked topics was 204° (95% CI = [197°, 211°]), placing it squarely in the aloof-introverted octant; the circular mean of female-topics was 359° (95% CI = [6°, 352°]), aligned with the affiliation axis and in the warm-agreeable octant. Due to the wider spread of male-linked topics, both the mean and variability of the gender-linked topics were needed to accurately characterize their distributions. Male-linked topics were more variable, reflected by the wider shaded regions (±1 unit of variance) around the mean in Fig 6.

### Discussion

Our labeling method automatically labeled affiliative and assertive topics of language. Affiliative, interpersonally warmer language was used more often by female participants. Contrary to past research [79] and popular stereotypes [80, 81], we did *not* find clear gender differences in assertive language. Instead, we found that male participants were more likely to use language that was both highly assertive *and* colder (e.g., swearing, criticism, controversial topics), while women were more likely to use language that was highly assertive but also warmer (e.g., expressions of positive emotion and warmth towards others). While average gender differences in assertive language were small, male-linked language in assertiveness was more variable. While some male-linked topics were cold and assertive, others were cold yet highly *un*assertive. These unassertive topics contained relatively neutral language about objects (e.g., computers, films, music, video games). Ultimately, the greatest distinction between female- and male-linked language was in terms of the level of affiliation and interpersonal warmth.

Placing language into interpersonal space revealed similarities between topics that were not obvious from a direct analysis of their words. Consider the topic containing the words “opinion”, “opinions”, “logic”, “based”, “political”, and “fact”. This topic was among the most male-linked (*d* = .40) and falls in the aloof-introverted octant. Neighbors of this topic in interpersonal space included topics about government and taxes, knives and stabbing, and death. While they are diverse in semantic content, they share the same aversive interpersonal style and are all potentially unsettling topics in an informal public social setting like Facebook. They were also used far more by male than female participants. On the other hand, the largest cluster of strongly female-linked topics in the gregarious-extraverted octant was loaded with positive evaluations and expressions about friends and families.

## General Discussion

We explored the linguistic features that account for gender differences in language use. In Study 1, our open-vocabulary method identified hundreds of topics that were used significantly more by one gender. Although the effect size of gender difference for most topics was small, each topic represents a single dimension in the high-dimensional construct of language. Because topics are not perfectly correlated, small group differences across many single dimensions aggregate to create much larger differences in multidimensional space. However, the goal of our study was not to simply demonstrate that substantial gender language differences exist, but to describe and provide some psychological insight into the psychological patterns of these differences.

In Study 2, our psychological labeling method revealed that gender differences were largely confined to differences in affiliative language. We found a surprising degree of gender similarity in assertive language. The former finding is consistent with several studies, but the latter is at odds with past research and with gender stereotypes regarding assertiveness. Commonly held stereotypes often portray men as more assertive and cold, while characterizing women as more passive and nurturing [82, 83].

One explanation for our finding of gender similarity in assertiveness may be found in social role theory [84], which holds that the disproportionate allocation of men and women into different social roles contributes to gender specific behavior. For example, men are more likely to hold supervisory positions (e.g., physicians, organizational leaders) and women are more likely to hold supervisee positions (e.g., nurses, supervisees). These positions have corresponding expectations of assertive and affiliative behavior. Observed gender differences in behavior are partially confounded with the social roles that men and women are more likely to hold. From this perspective, there should be no gender differences among men and women in similar social roles (e.g., among male and female leaders). Supporting this prediction, Moskowitz, Suh, and Desaulniers [85] tracked interactions with supervisors, co-workers, and supervisees, and found that these social roles–not gender–predicted assertive behavior. When in supervisory roles, men and women were equally assertive.

The online network environment may act as a social equalizer, placing users at different power levels into similar social roles–everyone is a “friend”. Status messages can be viewed by all members of one’s social network. These factors may decrease the salience of gender roles in online contexts that create differences in assertive and submissive behavior in other situations. Therefore, social role theory may not explain the gender differences we found in affiliative language. In fact, our findings are consistent with a large body of evidence detailing gender differences in affiliative expression [86], including smiling [87], disclosing and referencing emotions when engaging others [88, 89], and expressing agreement and warmth [90, 91, 92]. The gender differences in affiliative expression may be consistent with evolutionary perspectives of females as more invested in forming social bonds [93], perhaps suggesting that such biological differences extend to the modern online environment.

The labeling method applied in this study offers a useful tool for linguistic analyses in general. Because topics were labeled automatically, our computational method avoids rater biases that might enter the labeling process with hand-labeling features. For example, if a topic seems male-typical, raters may subconsciously rate it as more assertive or less affiliative, due to their own underlying stereotypes. This same approach could be extended to characterize language along other dimensions to test a wider range of hypotheses.

### Limitations

In this study, we limited our analyses to the dimensions of affiliation and assertiveness due to their prominence in gender differences research, but other dimensions of language could be considered using this labeling method. For example, language topics could be mapped to dimensions such as talkativeness [94, 95] or self-referencing [96]. Future work could also investigate gender differences in other psychological correlates of specific language features. Follow-up studies using alternative and more fine-grained analysis of assertive and affiliative language are also warranted. Calculations for assertiveness and affiliation were based on DeYoung et al.’s conversion. Alternative conversions are possible, and testing the best angles on large samples, especially in the context of social media, are needed. Further, our finding that self-identified females were slightly more assertive than men may have been partly impacted by the definition of assertiveness used in the study. Indirect influence were counted as assertive language, but others might suggest that assertiveness only occurs through direct means. Future work might examine the difference between indirect and direct aspects of assertiveness, and gender might moderate any differences that occur.

A potential limitation of this study is that all language data was collected through the MyPersonality Facebook application, which differs from the context of previous gender language studies. Interests in taking personality tests and willingness to voluntarily share their status updates might be sources of selection bias. A more general concern is that behavior and self-presentation in online social networks may be different than offline contexts. While some research suggests that users accurately present themselves to their social network [97], self-presentation biases and unique aspects of the Facebook culture may have influenced the results. Social media is a continually evolving context, and the extent to which findings generalize to offline contexts is an open question [98], which should be examined in future work.

While social media users are younger than the general population [99], most participants in our sample were slightly older than those from a typical undergraduate sample (median ages were 23 and 22) and a quarter of the sample was in their late 20s or older. Thus, our social media sample is on par or more diverse than the samples used in most research in this area in terms of age [100, 101].

Despite the limitations of social media samples, they allow researchers to study questions at on a much larger scale than is typically possible. The total sample size afforded by social media in our two studies (*N* = 68,228) was roughly an order of magnitude larger than the combined sample size across all studies included in Leaper and Ayres’ (2007) meta-analyses of gender language differences (*n*s ranged from 2,541 to 4,385, each combining 50–70 studies). Finally, effect sizes were small by conventional standards, although they were similar in size to other studies of language on social media. The large sample size provides power to detect small effects, but the practical meaning of small effects, especially in other contexts or for single users is unclear, and the results should be interpreted accordingly.

## Conclusion

In a large study of gender and language, we found that men and women use language differently, with the greatest difference being in the degree of interpersonal warmth. The language most characteristic of self-identified females was warmer, friendlier, and focused on people, whereas self-identified males’ most characteristic language was more socially distant, disagreeable, and focused on objects. Contrary to expectations, women used slightly more assertive language than men. We found affiliative and assertive language through established assessments rather than human judgments, the latter of which are more prone to rater-bias. Our approach borrows equally from computational linguistics and psychological theory, and we propose that similar interdisciplinary approaches may be useful for seeing old psychological questions in a new light.
